# Supplementary material for: Bicycle balance assist system reduces roll and steering motion for young and older bicyclists during real-life safety challenges
Source: PeerJ. 2023 Oct 17;11:e16206. doi: 10.7717/peerj.16206 (PMC10588700; doi:10.7717/peerj.16206)
Supplement: Supplemental Information 1 [file peerj-11-16206-s001.pdf]

1 APPENDIX 1

1.1 Speed

Since motion variability is expected between rides at 2 m/s and at 5 m/s with the same rider, we performed post-hoc ANOVA analysis to confirm the subjects followed the instruction and kept the speed constant. The analysis confirmed the constant speed assumption as we found that there were no significant differences in average ($p > 0.05$), standard deviation ($p > 0.05$), and range of speed (maximum-minimum speed, $p > 0.05$) between trials per subject.

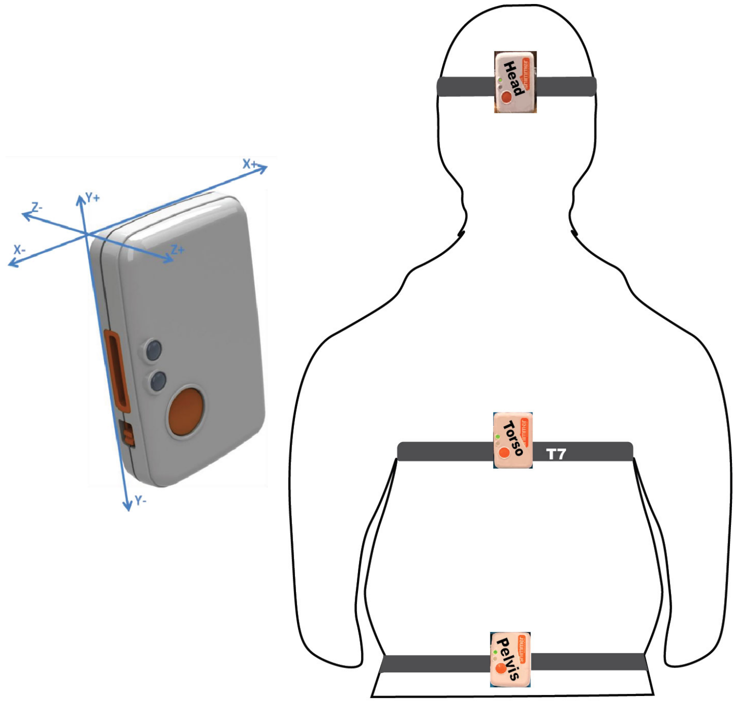

Figure 1. Shimmer3 IMU (3 axis) sensors placement on one young participant. The data from the torso was used in the analysis.

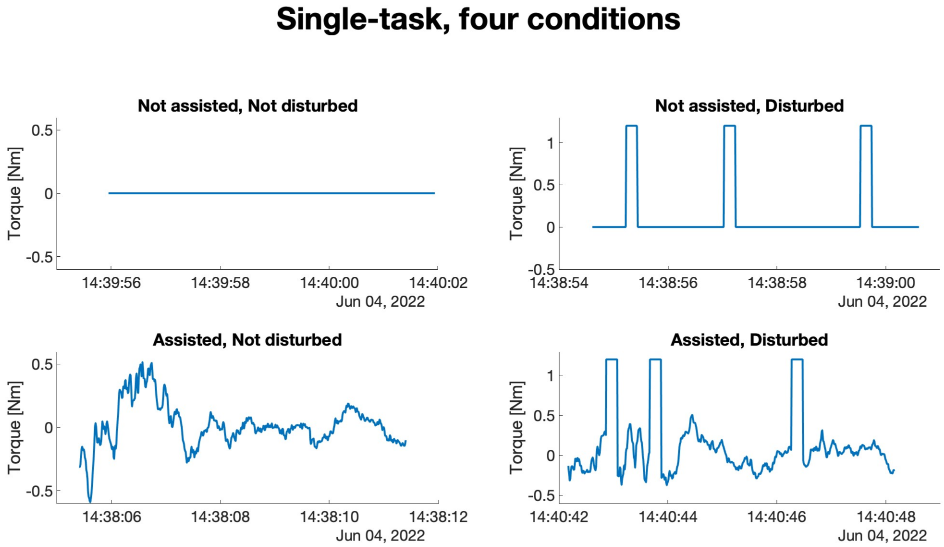

Figure 2. Demonstration of the implementation of four different conditions: Not Assisted Not Disturbed, Not Assisted Disturbed, Assisted Not Disturbed, Assisted Disturbed. Assisted implies the balance assist system was activated and the steer assistive torque was applied. Disturbed implies that the disturbances were applied.

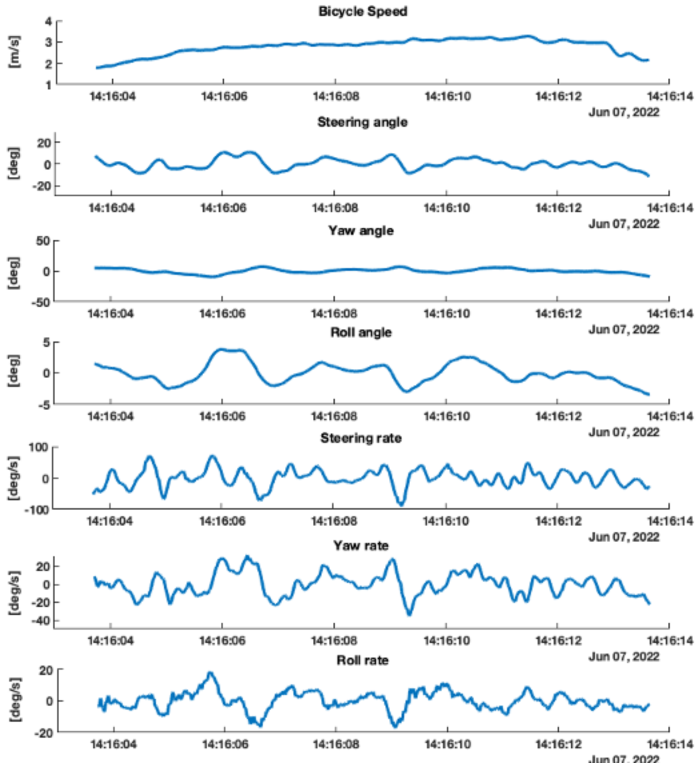

Figure 3. Segmented part of the time series starting at 1.5 m/s forward speed (after accelerating from the starting point). The figure depicts the cycling in a steady-state phase with an approximately constant speed until the rider either decelerates and reaches 1.5 m/s or begins to turn to return to the starting point, as indexed by a yaw rate $>$ 4 deg/s.

1.2 Statistical analysis on the standard deviation of the outcome measures

In this section, we present the analysis results for the standard deviation of the roll rate and the steer rate in single- and multi-task cycling. The analysis was performed for all subjects, and we observed the following consistent findings with the mean magnitude roll rate and steer rate:

Single-task

Standard deviation of Roll rate

Within Subjects Effects

Cases	Sum of Squares	df	Mean Square	F	p
Balance assist	13.473	1	13.473	44.375	< .001
Balance assist ∗ agegroup	0.484	1	0.484	1.593	0.217
Residuals	9.108	30	0.304		
Disturbance	6.673	1	6.673	12.877	0.001
Disturbance ∗ agegroup	0.444	1	0.444	0.857	0.362
Residuals	15.546	30	0.518		
Balance assist ∗ Disturbance	0.027	1	0.027	0.163	0.689
Balance assist ∗ Disturbance ∗ agegroup	0.083	1	0.083	0.490	0.489
Residuals	5.055	30	0.169		

Note. Type III Sum of Squares

Between Subjects Effects

Cases	Sum of Squares	df	Mean Square	F	p
agegroup	23.385	1	23.385	6.550	0.016
Residuals	107.098	30	3.570		

Note. Type III Sum of Squares

Standard deviation of Steer rate

Within Subjects Effects

Cases	Sum of Squares	df	Mean Square	F	p
Balance assist	113.093	1	113.093	30.081	< .001
Balance assist ∗ agegroup	2.889	1	2.889	0.768	0.388
Residuals	112.789	30	3.760		
Disturbance	558.555	1	558.555	34.150	< .001
Disturbance ∗ agegroup	20.122	1	20.122	1.230	0.276
Residuals	490.675	30	16.356		
Balance assist ∗ Disturbance	8.190	1	8.190	1.786	0.191
Balance assist ∗ Disturbance ∗ agegroup	6.813	1	6.813	1.486	0.232
Residuals	137.550	30	4.585		

Note. Type III Sum of Squares

Between Subjects Effects

Cases	Sum of Squares	df	Mean Square	F	p
agegroup	271.987	1	271.987	2.294	0.140
Residuals	3557.037	30	118.568		

Note. Type III Sum of Squares

Multi-task

Standard deviation of Roll rate

Within Subjects Effects

Cases	Sum of Squares	df	Mean Square	F	p
Balance assist	8.078	1	8.078	21.770	< .001
Balance assist ∗ agegroup	0.041	1	0.041	0.111	0.742
Residuals	9.648	26	0.371		
Disturbance	77.922	1	77.922	65.740	< .001
Disturbance ∗ agegroup	2.924	1	2.924	2.467	0.128
Residuals	30.818	26	1.185		
Balance assist ∗ Disturbance	0.321	1	0.321	0.896	0.353
Balance assist ∗ Disturbance ∗ agegroup	1.051	1	1.051	2.934	0.099
Residuals	9.309	26	0.358		

Note. Type III Sum of Squares

Between Subjects Effects

Cases	Sum of Squares	df	Mean Square	F	p
agegroup	7.336	1	7.336	2.046	0.165
Residuals	93.224	26	3.586		

Note. Type III Sum of Squares

Standard deviation of Steer rate

Within Subjects Effects

Cases	Sum of Squares	df	Mean Square	F	p
Balance assist	64.786	1	64.786	9.760	0.004
Balance assist ∗ agegroup	2.736	1	2.736	0.412	0.527
Residuals	172.594	26	6.638		
Disturbance	1948.894	1	1948.894	116.161	< .001
Disturbance ∗ agegroup	12.095	1	12.095	0.721	0.404
Residuals	436.217	26	16.778		
Balance assist ∗ Disturbance	0.049	1	0.049	0.008	0.928
Balance assist ∗ Disturbance ∗ agegroup	9.944	1	9.944	1.693	0.205
Residuals	152.730	26	5.874		

Note. Type III Sum of Squares

Between Subjects Effects

Cases	Sum of Squares	df	Mean Square	F	p
agegroup	235.313	1	235.313	3.064	0.092
Residuals	1996.544	26	76.790		

Note. Type III Sum of Squares
